# A Lymphoid-Enriched Spatial Immune Niche Defines an Immunotherapy-Responsive State in Hepatocellular Carcinoma

**DOI:** 10.3390/cancers18213442

**Published:** 2026-10-24

**Authors:** Mingxiu Cai, Kaiheng Hu, Haichang Wang, Jing Li, Yiqi Wu, Qirui Han, Jixuan Chen, Wei Zhong, Xiangfan Huang, Fangyuan Ding, Yuan Du, Yiming Liu, Feng Zhang

**Affiliations:** 1 Jiangxi Province Key Laboratory of Bioengineering Drugs, Institute of Translational Medicine, Jiangxi Medical College, Nanchang University, Nanchang 330031, China; m.cai@se23.qmul.ac.uk (M.C.);; 2 Queen Mary School, Nanchang University, Nanchang 330031, Chinajp4217123123@qmul.ac.uk (H.W.);; 3 College of Life Science, Jiangxi Science and Technology Normal University, Nanchang 330032, China; 4 Hepatobiliary Surgery, Department of General Surgery, Cancer Metastasis Institute, Huashan Hospital, Fudan University, Shanghai 200437, China; 5 The First Clinical Medical College, Nanchang University, Nanchang 330031, China; 6 The Second Clinical Medical College, Nanchang University, Nanchang 330031, China; 7 Jiangxi Provincial People’s Hospital (The First Affiliated Hospital of Nanchang Medical College), Nanchang 330006, China

**Keywords:** hepatocellular carcinoma, immunotherapy, spatial transcriptomics, tumour microenvironment

## Abstract

Background: Atezolizumab plus bevacizumab is a first-line therapy for advanced hepatocellular carcinoma (HCC), yet only a subset of patients achieve durable benefit. The spatial organization of the tumour microenvironment (TME) and its relationship to immunotherapy response remain incompletely understood. Methods: We integrated single-cell transcriptomics, spatial transcriptomics, spatial proteomics, and bulk RNA-sequencing cohorts to characterize spatially organized immune ecosystems associated with immunotherapy response in HCC. Single-cell RNA sequencing from 100 HCC samples was used to construct a cellular reference, which was integrated with spatial transcriptomics from 12 sections to identify nine consensus spatial niches. Results: Niche 9 represented a lymphoid-enriched immune state characterized by B cells, plasma cells, natural killer cells, T-cell subsets, macrophages, and tertiary lymphoid structure-associated transcriptional features. Independent validation in a pretreatment, response-annotated Visium cohort showed enrichment of Niche 9-associated transcriptional activity in immunotherapy responders, while spatially co-registered 51-plex CODEX profiling provided orthogonal protein-level support for its lymphoid and immune-enriched phenotype. Multi-omics characterization further revealed coordinated immune-cell localization, ligand–receptor-associated patterns, and distinct metabolic programmes, with MEI1-positive plasma cells displaying enhanced secretory and stress-adaptation states. A nine-gene transcriptional signature derived from Niche 9 was associated with atezolizumab-plus-bevacizumab response, enhanced lymphoid immune features, lower Tumour Immune Dysfunction and Exclusion scores, and favourable clinical outcomes in independent cohorts. Conclusions: These multi-cohort and multi-omics analyses define a spatially derived lymphoid-enriched immune programme and link its molecular features to immunotherapy sensitivity in HCC.

## Simple Summary

Immunotherapy has improved treatment options for patients with advanced liver cancer, but not all patients benefit equally. Understanding how immune cells are organized within tumours may help explain these differences and identify patients more likely to respond to treatment. In this study, we combined several molecular profiling approaches to investigate the spatial organization of immune cells in liver cancer. We identified a distinct immune-rich region, termed Niche 9, characterized by the enrichment of B cells, T cells, and other immune populations. The molecular features associated with this region were linked to immunotherapy response and favourable clinical outcomes across independent patient cohorts. These findings provide insights into how the organization of immune cells relates to treatment response and highlight the potential of spatially derived molecular markers to support future research on personalized immunotherapy for liver cancer.

## 1. Introduction

Primary liver cancer is the sixth most commonly diagnosed malignancy worldwide and the third leading cause of cancer-related mortality [1,2]. Hepatocellular carcinoma (HCC), the predominant histological subtype, accounts for approximately 80–85% of primary liver cancers and is frequently diagnosed at intermediate or advanced stages, contributing to poor clinical outcomes [3,4].

Treatment of HCC is guided by tumour stage and liver function [5]. While selected early-stage patients may benefit from surgery, transplantation, or local ablation, postoperative recurrence remains a major clinical challenge [6]. Advanced HCC is primarily managed with systemic therapy, with immune checkpoint inhibitors (ICIs) substantially reshaping the therapeutic landscape [7]. But their efficacy remains constrained by the complex tumour microenvironment (TME), including immune exclusion, aberrant angiogenesis, hypoxia, vascular dysfunction, and immunosuppressive cellular states. VEGF-mediated abnormalities further impair immune infiltration and promote tumour immune evasion [8,9]. Accordingly, atezolizumab plus bevacizumab (ATZ/BVZ), combining immune checkpoint blockade with anti-angiogenic therapy, has become a standard first-line treatment for advanced HCC [10,11,12]. Nevertheless, durable benefit is limited to a subset of patients, highlighting the need to define microenvironmental features associated with therapeutic response [13].

The TME comprises malignant, immune, stromal, and vascular compartments whose spatial organization can influence tumour progression and treatment sensitivity [14]. Beyond immune-cell abundance alone, locally organized immune states may contribute to effective anti-tumour immunity. Tertiary lymphoid structure (TLS)-associated features represent one form of such immune organization and have been linked to improved responses to immune checkpoint blockade [15,16,17]. However, whether reproducible spatial immune states exist across independent HCC cohorts and retain consistent cellular and protein-level characteristics remains unclear.

Previous studies investigating HCC immunotherapy response have primarily relied on bulk RNA sequencing, single-cell RNA sequencing, and conventional experimental approaches [18,19]. Although these approaches have revealed substantial cellular heterogeneity and immune-related mechanisms, they cannot fully resolve the spatial organization of tumour ecosystems or context-dependent cellular interactions [20,21]. Spatial transcriptomics preserves tissue architecture while enabling transcriptome-wide characterization of local microenvironments, allowing the identification of spatial niches with distinct cellular compositions, molecular programmes, and functional states [22,23,24,25]. Such spatially defined immune niches may represent important organizational units that reflect local immune activity and generate transcriptional programmes associated with therapeutic sensitivity [26,27]. However, spatial immune niches and their associated molecular programmes in relation to ATZ/BVZ-treated HCC remain insufficiently characterized.

Here, we integrated single-cell transcriptomics, spatial transcriptomics, spatial proteomics, and bulk RNA-sequencing cohorts to characterize spatially organized immune ecosystems in HCC. We first combined a large single-cell reference with spatial transcriptomic data to define immune-enriched spatial states and their associated molecular programs. We then evaluated the Niche 9-associated immune state in an independent response-annotated pretreatment spatial multi-omics cohort and used spatially co-registered proteomic profiling to provide orthogonal cellular and protein-level validation. Finally, we derived a spatially informed transcriptional program and assessed its association with immunotherapy response and clinical outcomes across independent cohorts, including patients treated with atezolizumab plus bevacizumab.

## 2. Material and Methods

### 2.1. Data Collection

This study integrated one scRNA-seq dataset (PRJCA007744), one spatial transcriptomic discovery cohort (HRA000437), an independent response-annotated spatial multi-omics cohort, and three bulk RNA-sequencing immunotherapy cohorts (EGAS00001005503, GSE202069, and GSE285963) in hepatocellular carcinoma (HCC). PRJCA007744 comprised 100 samples from 79 patients and was used to construct the single-cell reference atlas. HRA000437 included 12 Visium sections from five patients, comprising four normal–tumour junction and eight tumour sections, and served as the spatial discovery cohort.

Independent spatial validation used a perioperative nivolumab-based cohort of 13 patients (six responders and seven nonresponders), including 22 Visium samples and 48 fields of view profiled with a 51-plex Akoya PhenoCycler panel. Pretreatment Visium samples were used to evaluate Niche 9 activity in relation to subsequent response, while spatial proteomic data provided orthogonal validation of its cellular and protein features.

For clinical validation, EGAS00001005503 comprised 304 ATZ/BVZ-treated patients and served as the primary cohort for response and survival analyses. GSE202069 provided independent immunotherapy validation, while GSE285963 included 87 pretreatment ATZ/BVZ samples used to validate niche-associated transcriptional signatures.

### 2.2. Processing of scRNA-Seq Data

scRNA-seq data were processed using Scanpy [28]. Cells with fewer than 500 detected genes, more than 30,000 unique molecular identifiers, or mitochondrial transcript proportions exceeding 50% were excluded, and genes expressed in fewer than three cells were removed. After normalization and log transformation, the top 3000 highly variable genes were selected. Principal component analysis was performed, and the top 50 components were used to construct the adjacency graph. Batch effects were corrected using BBKNN with sample identity as the batch variable [29]. Leiden clustering was performed at a resolution of 1.0, and major cell types and subpopulations were annotated using canonical marker genes. Observed-to-expected ratios were calculated to assess cell-type enrichment across pathological origins and clinical groups.

### 2.3. Processing of Spatial Transcriptomic Data

Spatial transcriptomic data were preprocessed following the workflow and parameter settings described by Wu et al. [30]. STAligner was used for spatial alignment and integration [31]. Tumour and normal regions were independently annotated by two experienced pathologists using QuPath (v0.7.0), and only regions with concordant annotations were retained.

### 2.4. Spatial Deconvolution and Niche Identification

The annotated HCC single-cell atlas was used as the reference for spatial deconvolution with cell2location to estimate cell-type abundance per spatial spot [32]. Leiden clustering was applied to the inferred abundance matrix at a resolution of 0.7, and highly similar subclusters were merged into consensus spatial niches by hierarchical clustering using a Euclidean distance threshold of 0.1. Pearson correlation analysis was used to assess inter-niche similarity.

Differentially expressed genes between niches were identified using the Wilcoxon rank-sum test, with fold change > 1.5 and Benjamini–Hochberg FDR < 0.05. The top 100 upregulated genes from each niche were used to define niche-associated transcriptional programmes, which were evaluated in independent bulk RNA-seq cohorts by GSVA. Patients were stratified by the median programme score, and associations with immunotherapy response, overall survival, and progression-free survival were assessed using Kaplan–Meier analysis and log-rank tests.

### 2.5. Independent Spatial Validation

The Niche 9-associated gene programme derived from the HRA000437 discovery cohort was projected onto pretreatment Visium samples from an independent response-annotated HCC spatial multi-omics cohort. Niche 9 activity was quantified using AUCell, and spots within the top 10% of AUCell scores were defined as Niche 9-high.

Spatial maps were generated to visualize Niche 9 activity in responder and nonresponder specimens. The proportions of Niche 9-high and non-high spots were summarized by response group and compared at the pooled spot level using Fisher’s exact test.

### 2.6. Orthogonal CODEX Validation of Niche 9

Processed 51-plex Akoya PhenoCycler data from the independent HCC spatial multi-omics cohort were used for orthogonal validation. Spatially co-registered Visium locations were linked to overlapping CODEX cells, restricting analyses to pretreatment, non-adjacent-liver regions with adequate coverage.

CODEX cell-type fractions were compared between Niche 9-high and other regions using two-sided Mann–Whitney U tests with Benjamini–Hochberg correction. Associations between continuous Niche 9 AUCell scores and cell-type fractions were assessed using within-region-centred Spearman correlation with FDR correction.

For protein-level validation, normalized marker intensities were averaged at each spatial location and compared between Niche 9-high and other regions. Cliff’s delta was used to quantify effect size. Region-centred protein values were further assessed using 5000 within-region label permutations with FDR correction. Proteins were considered enriched when the permutation FDR < 0.05; raw mean, median, Cliff’s delta, and region-centred differences were consistently positive; and Cliff’s delta was ≥0.147.

### 2.7. Functional Characterisation of Spatial Niches

Niche-associated pathway activity was assessed using over-representation analysis with decoupleR based on curated immune-related gene sets, including tertiary lymphoid structure-related signatures, B-cell activation, chemokine signalling, lymphocyte co-stimulation, and leukocyte adhesion. For the response-associated lymphoid-enriched niche, Gene Ontology and Kyoto Encyclopedia of Genes and Genomes enrichment analyses were performed using clusterProfiler 4.20.2. Metabolic fluxes and balances were inferred from spatial transcriptomic data using scFEA v1.0 [33], and differential metabolic features between the Niche9 and other niches were assessed using Cliff’s delta with false-discovery-rate adjustment and sample-level mean differences.

### 2.8. Spatial Co-Localisation and Ligand–Receptor Interaction Analysis

Spatial co-localisation relationships within and adjacent to the response-associated niche were evaluated using the MISTy framework in mistyR [34]. Intra-view results were used to estimate local co-localisation patterns between major cell types, and normalized importance scores were used to quantify spatial dependencies. Ligand–receptor interactions within spatial niches were analysed using stLearn 1.4.1 [35]. Interaction strength and significance were assessed across spatial spots, with statistical significance determined by permutation testing using 10,000 randomly generated gene pairs.

### 2.9. Transcription Factor Regulon Analysis

Transcriptional regulatory programs in MEI1-positive plasma cells were analysed using pySCENIC [36]. Human transcription factor annotations, motif-to-transcription-factor mappings, and the hg38 cisTarget ranking database were used as references. Regulon activity was quantified using AUCell scores, and differential regulon activity between MEI1-positive and MEI1-negative plasma cells was assessed using the Wilcoxon rank-sum test. Genes from significantly altered regulons were further subjected to Reactome and WikiPathways enrichment analyses.

### 2.10. Machine Learning Model Construction and Interpretation

The top 100 upregulated genes from immunotherapy response-associated spatial niches were selected as candidate predictive features. Nine machine-learning algorithms were evaluated: ElasticNet_Logistic, ExtraTrees, KNN, LASSO_Logistic, Linear_SVM, PLS_Logistic, RBF_SVM, Ridge_Logistic, and XGBoost. The EGAS00001005503 cohort was stratified into training (70%) and internal validation (30%) sets, while GSE202069 served as an independent external validation cohort. To minimize overfitting, repeated nested cross-validation was performed exclusively within the training set, comprising five outer folds repeated ten times and five inner folds for hyperparameter optimization using GridSearchCV. ROC-AUC was used as the primary optimization metric, with all supervised feature selection and hyperparameter tuning restricted to the corresponding training folds to prevent data leakage. Models were compared based on their mean outer-fold ROC-AUC. For the selected XGBoost model, feature selection was performed using gain-based importance, followed by fivefold stratified cross-validation with randomized hyperparameter search (20 iterations), optimizing tree complexity, learning rate, subsampling, and regularization parameters. Class imbalance was addressed through stratified sampling and class-weight adjustment. The optimal classification threshold was determined using the Youden index based on cross-validated training predictions. The finalized model was evaluated using ROC-AUC, accuracy, precision, recall, and F1 score, with ROC-AUC and 95% confidence intervals reported for the internal and external validation cohorts. SHAP analysis was performed to quantify individual feature contributions and interpret model predictions.

### 2.11. Association Between Model Signature and Immunotherapy Response

Based on feature importance rankings and Shapley additive explanations from the optimal XGBoost model, nine core genes were selected to construct a response-associated signature. Gene set variation analysis was used to calculate signature scores in EGAS00001005503 and GSE285963. Patients were stratified into high- and low-signature groups by the median score, and the clinical relevance of the signature was evaluated by associations with immune-cell infiltration, tertiary lymphoid structure-related signatures, immune activation gene sets, and Tumour Immune Dysfunction and Exclusion scores.

## 3. Results

### 3.1. Single-Cell Atlas Delineates the Cellular Landscape of HCC

**Figure 1:**
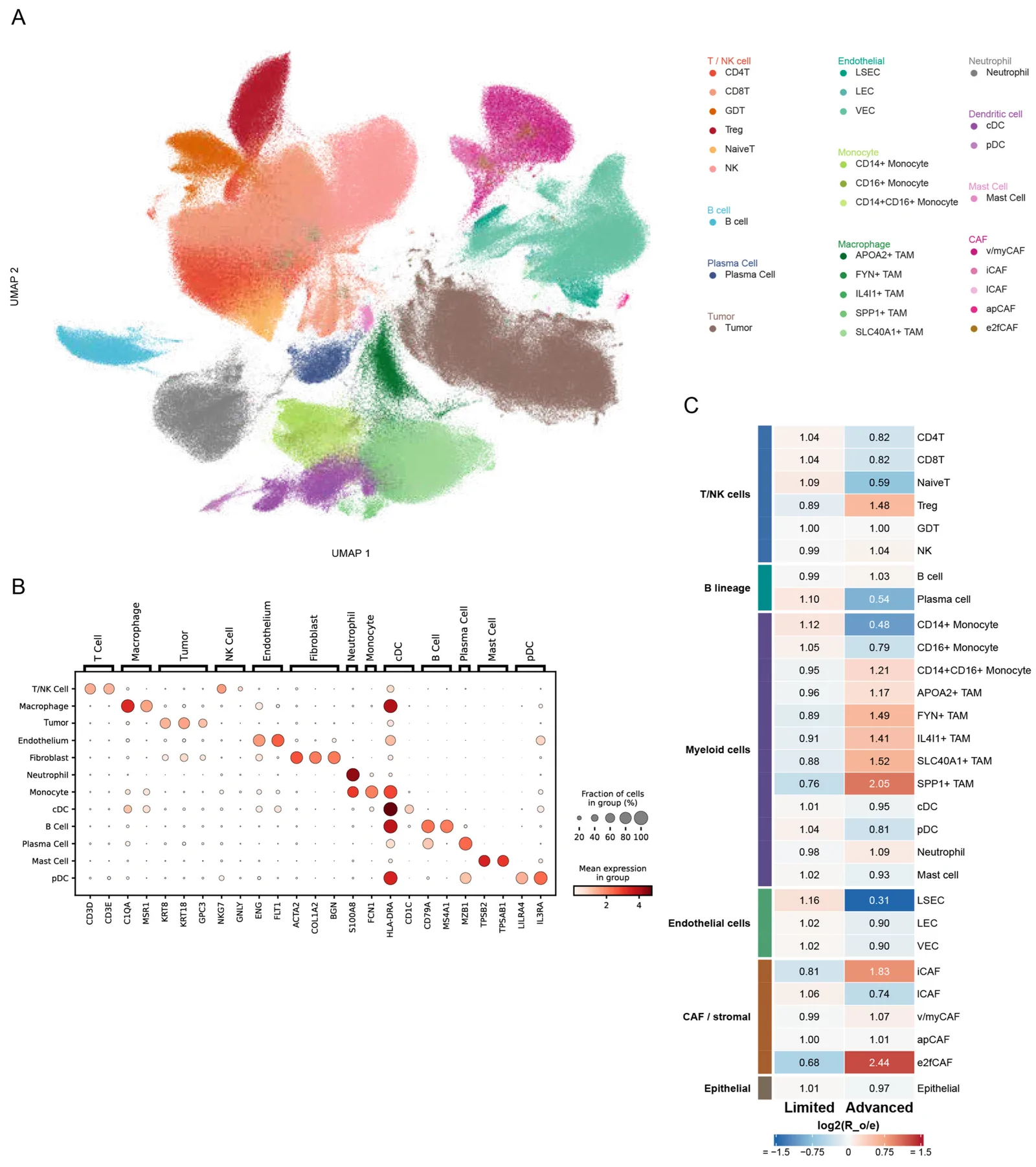
Single-cell transcriptomic landscape of hepatocellular carcinoma. (**A**) Uniform manifold approximation and projection of 871,457 cells from 79 patients, coloured by 13 major cell types and 29 secondary cell types. (**B**) Dot plots showing representative marker genes used for major cell-type annotation. (**C**) Heatmap of observed-to-expected ratios showing secondary cell-type enrichment across limited and advanced tumour stages.

We integrated 100 HCC scRNA-seq samples from PRJCA007744 and retained 871,457 high-quality cells after quality control. Batch effects were effectively corrected using BBKNN (Supplementary Figure S1A). Based on canonical markers, we identified 13 major cell types spanning immune, stromal, endothelial, and malignant compartments (Figure 1A,B), with further high-resolution reclustering shown in Supplementary Figure S1B–F. Ro/e enrichment analysis revealed stage-associated microenvironmental differences (Figure 1C). Early-stage tumours were enriched in naive T cells, plasma cells, and LSECs, whereas advanced tumours showed increased stromal and immunosuppressive populations, including e2fCAFs, SPP1-positive TAMs, and iCAFs.

### 3.2. Spatial Transcriptomics Identifies an Immune-Enriched Niche Linked to Immunotherapy Benefit

**Figure 2:**
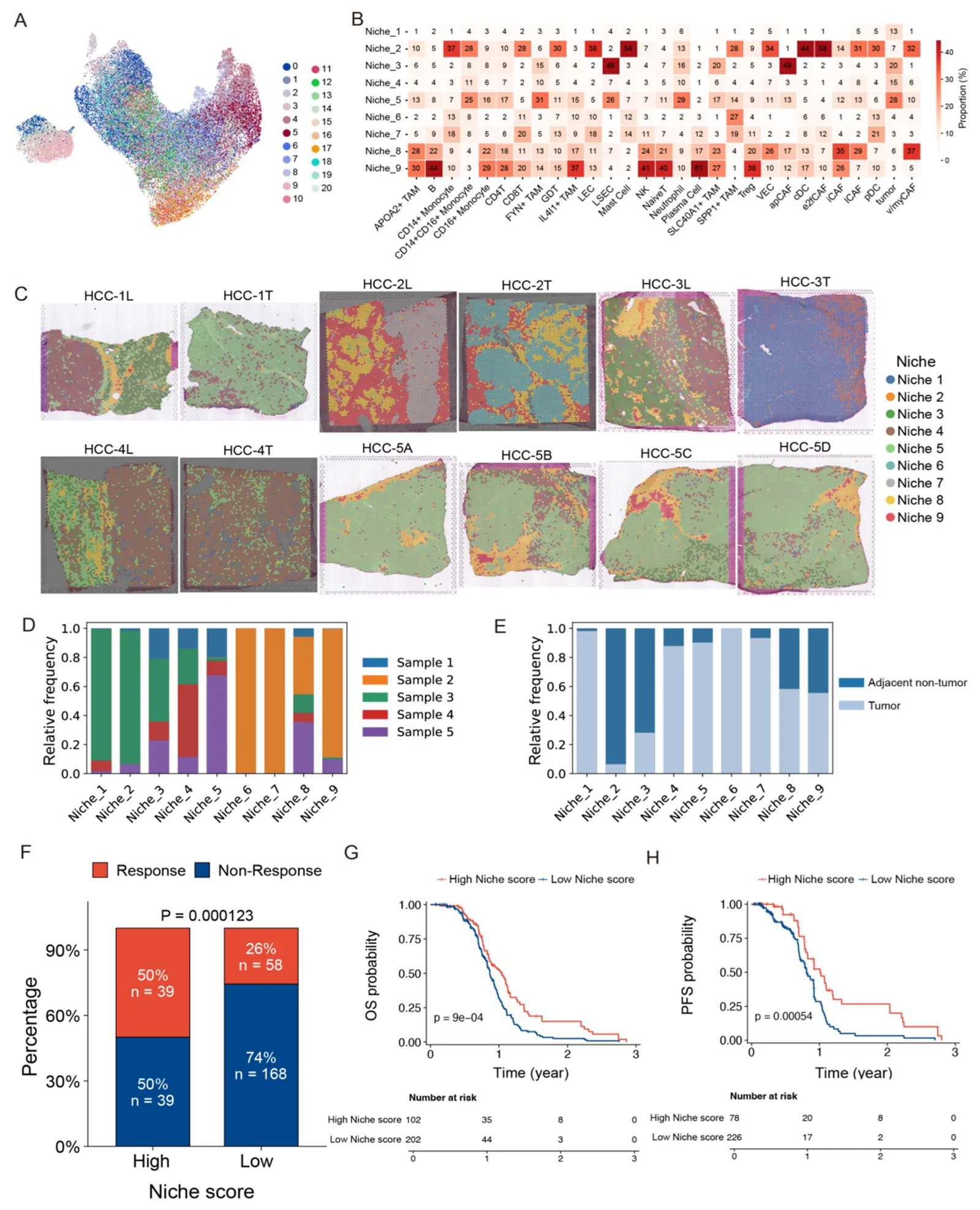
Identification of spatial niches and their clinical relevance in hepatocellular carcinoma. (**A**) Uniform manifold approximation and projection of integrated spatial transcriptomic spots from 12 hepatocellular carcinoma sections, coloured by cluster identity. (**B**) Heatmap showing the composition and enrichment of major cell types across spatial niches. (**C**) Spatial distribution of niches across different hepatocellular carcinoma sections. (**D**) Stacked bar plot showing the sample composition of each niche. (**E**) Stacked bar plot showing the relative proportions of tumour and adjacent normal regions annotated by two pathologists within each niche. (**F**) Response rates in the immunotherapy cohort stratified by the Niche 9 signature. *p* values were determined by Fisher’s exact test. (**G**,**H**) Kaplan–Meier curves of overall survival and progression-free survival in the immunotherapy cohort stratified by the Niche 9 signature.

We integrated 12 spatial transcriptomic sections comprising 48,416 spots and 33,538 genes. Cell2location-based deconvolution followed by clustering identified 21 initial spatial clusters, which were consolidated into nine consensus spatial niches based on hierarchical clustering and inter-cluster correlations (Figure 2A–C; Supplementary Figure S2A,B). These niches showed distinct tumour, normal-liver, myeloid, inflammatory, and stromal compositions.

Niche 9 displayed the strongest immune-enriched phenotype, with increased B cells, plasma cells, NK cells, naive T cells, regulatory T cells, and IL4I1-positive macrophages (Figure 2B). It was detected across multiple sections and distributed in both peritumoural and tumour regions (Figure 2C–E). Genes upregulated in Niche 9 were used to generate a niche-associated transcriptional programme, which was evaluated in independent bulk immunotherapy cohorts. Higher programme scores were associated with increased ATZ/BVZ response rates and prolonged overall and progression-free survival (Figure 2F–H), whereas several other niche-derived programmes showed distinct or opposite clinical associations (Supplementary Figure S2C–H).

### 3.3. Independent Spatial Validation Reproduces the Niche 9-Associated State

The top 100 Niche 9-upregulated genes from the discovery cohort were projected onto pretreatment Visium samples from an independent response-annotated HCC cohort. AUCell analysis revealed heterogeneous spatial distributions of Niche 9 activity, and spots within the top 10% of scores were defined as Niche 9-high (Supplementary Figure S3A).

At the pooled spot level, Niche 9-high regions were more frequent in responders than nonresponders (594/4013, 14.80% vs. 530/7227, 7.33%), corresponding to approximately twofold enrichment (Fisher’s exact test, OR = 2.20, *p* = 4.06 × 10^−35^; Supplementary Figure S3B). Because this analysis was performed at the pooled spot level, it was interpreted as supportive spatial evidence rather than patient-level predictive validation.

Spatially co-registered CODEX analysis further supported a lymphoid-enriched phenotype. Continuous Niche 9 activity correlated positively with several lymphoid and immune populations, including CD4 T cells, B cells, CD8 T cells, dendritic cells, and IFNγ-positive cells, and negatively with endothelial and epithelial populations (Supplementary Figure S3C). Protein-level analysis similarly showed enrichment of lymphoid, B-cell/TLS-associated, cytotoxic, antigen-presentation, and immune-regulatory markers in Niche 9-high regions (Supplementary Figure S3D).

### 3.4. Functional and Metabolic Features of the Immune-Enriched Niche

**Figure 3:**
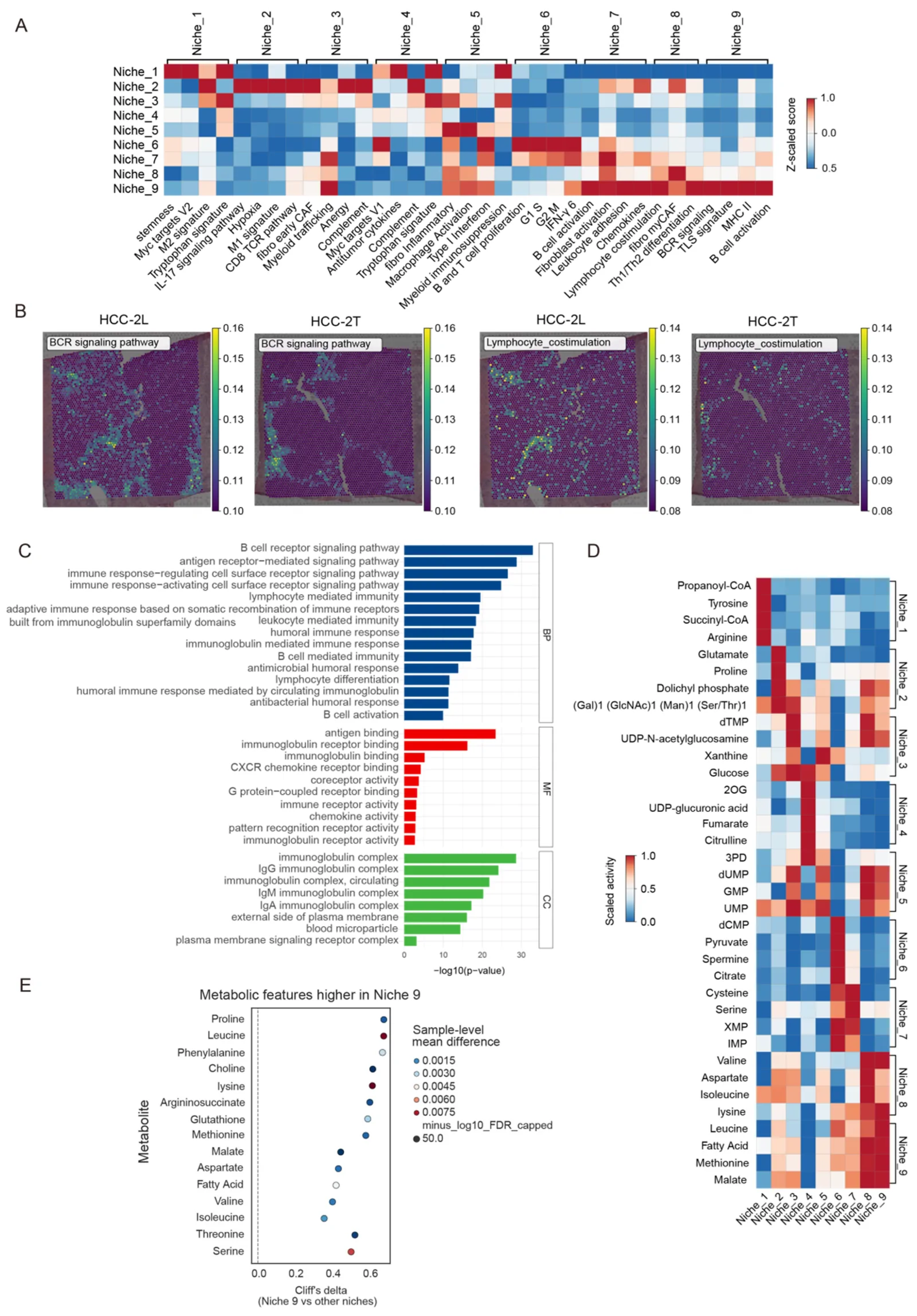
Functional and metabolic features of the lymphoid-enriched immune niche. (**A**) Heatmap showing immune pathway activity scores across spatial niches calculated using decoupleR. (**B**) Spatial visualization of B-cell receptor signalling and lymphocyte co-stimulation scores in representative sections. (**C**) Gene Ontology enrichment analysis of genes upregulated in Niche 9. (**D**) Heatmap of metabolite balance across spatial niches. (**E**) Dot plot of metabolic features enriched in Niche 9, ranked by Cliff’s delta with false-discovery-rate-adjusted significance and sample-level mean differences.

Niche 9 showed the strongest enrichment of adaptive immune and organized lymphoid programmes, including B-cell receptor, chemokine, lymphocyte co-stimulation, and leukocyte adhesion pathways (Figure 3A–C). Spatial mapping confirmed localized enrichment of B-cell receptor signalling and lymphocyte co-stimulation within Niche 9 (Figure 3B). Spatial metabolic inference further identified distinct amino acid, lipid, glutathione, methionine, and related metabolic programmes in Niche 9 compared with other niches (Figure 3D,E).

### 3.5. Spatial Co-Localization and Ligand–Receptor-Associated Gene Co-Expression Patterns

**Figure 4:**
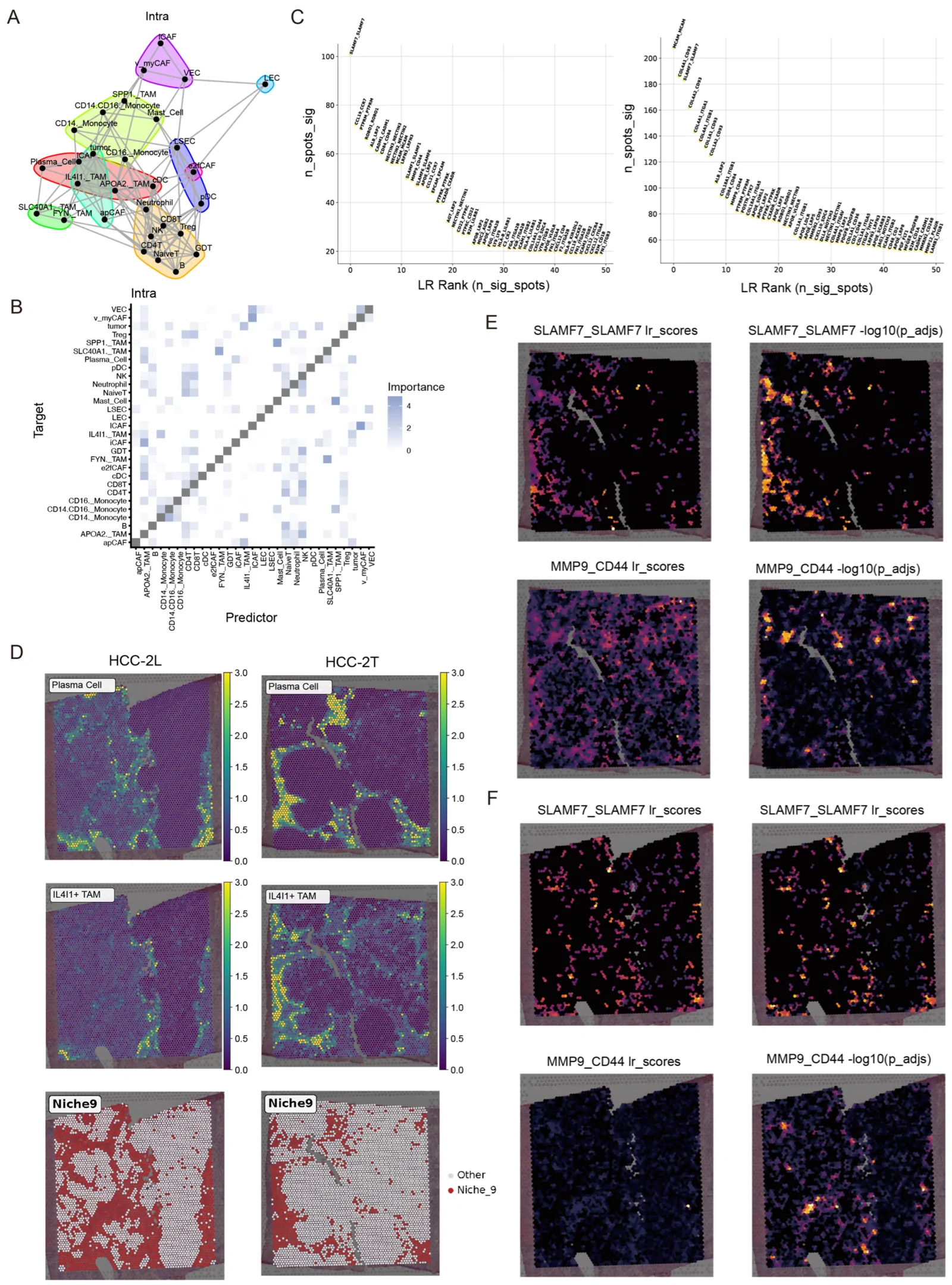
Spatial organization and ligand–receptor-associated patterns within the lymphoid-enriched immune niche. (**A**) Network of intra-niche cellular interactions within Niche 9. (**B**) Heatmap showing interaction importance between cell types in Niche 9. (**C**) Top ligand–receptor pairs in Niche 9 ranked by the number of spatial spots with significant signals. (**D**) Spatial distributions of plasma cells, IL4I1-positive tumour-associated macrophages, and Niche 9 regions in representative HCC-2L and HCC-2T sections. (**E**,**F**) Spatial maps of SLAMF7–SLAMF7 and MMP9–CD44 interaction scores and adjusted *p* values in representative sections.

MistyR analysis revealed co-localization among lymphoid, myeloid, plasma-cell, and tumour populations within Niche 9 (Figure 4A,B), while spatial mapping showed plasma cells and IL4I1-positive TAMs within and around Niche 9 regions (Figure 4D).

Spot-level co-expression analysis identified recurrent spatially enriched patterns, including SLAMF7–SLAMF7, CD84–CD84, CADM1–CADM1, and MMP9–CD44 (Figure 4C). Given the spot-based resolution, these were interpreted as spatial ligand–receptor-associated patterns rather than direct cell-type-specific interactions. SLAMF7–SLAMF7 and MMP9–CD44 showed localized enrichment in representative sections (Figure 4E,F). Analysis of SLAMF7–SLAMF7-enriched regions identified 145 shared upregulated genes enriched in immunoglobulin-associated components (Supplementary Figure S4A,B), and SLAMF7, CD44, and MMP9 were further evaluated in independent immunotherapy cohorts (Supplementary Figure S4C,D).

### 3.6. MEI1-Positive Plasma Cells Display a Distinct Secretory Transcriptional State

**Figure 5:**
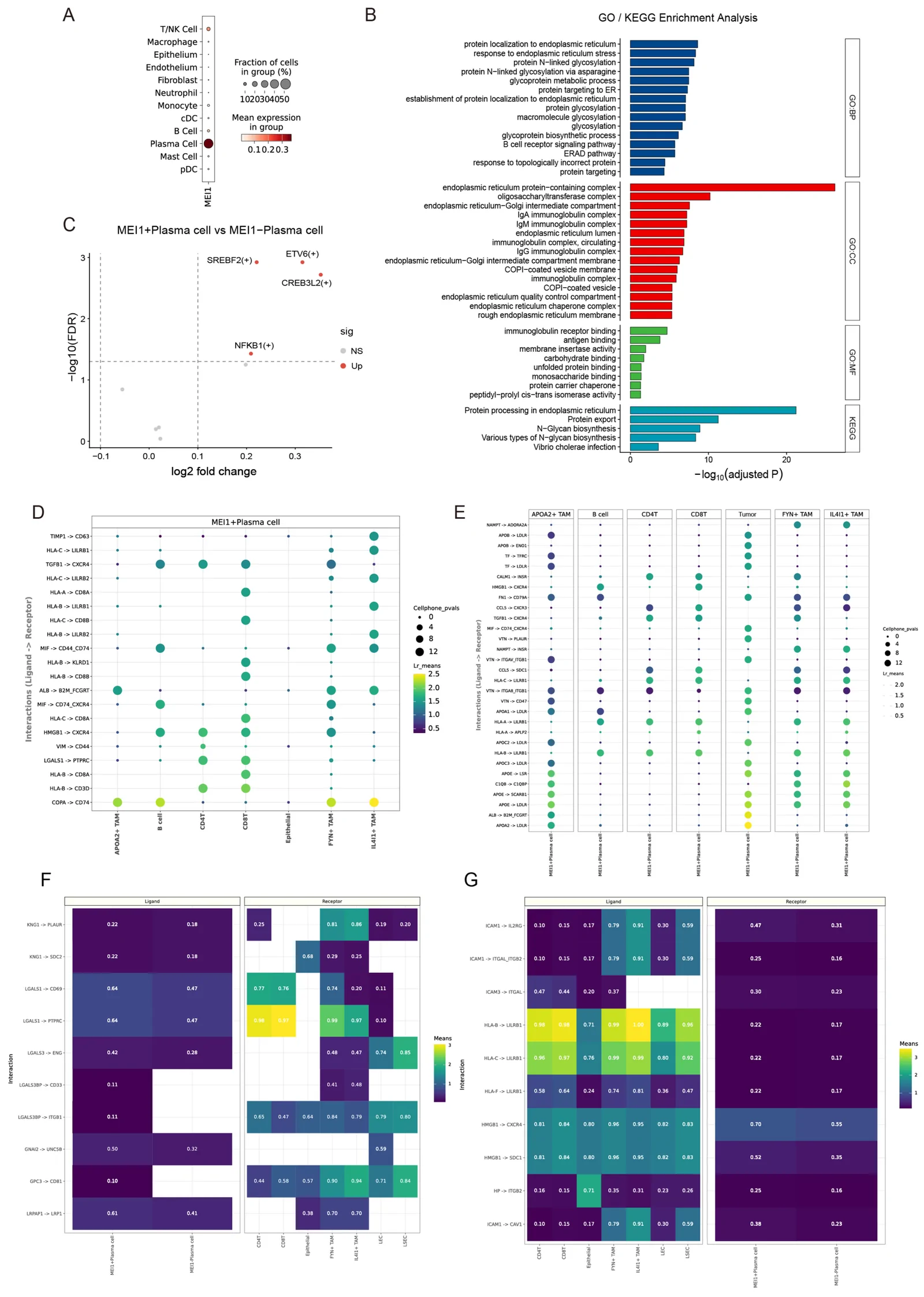
Functional, metabolic, and communication features of MEI1-positive plasma cells in hepatocellular carcinoma. (**A**) Dot plot showing MEI1 expression among major cell types. (**B**) Gene Ontology and Kyoto Encyclopedia of Genes and Genomes enrichment analysis of genes upregulated in MEI1-positive plasma cells. (**C**) Volcano plot showing differentially activated transcription factors between MEI1-negative and MEI1-positive plasma cells. (**D**,**E**) Dot plots showing ligand–receptor interactions involving MEI1-positive plasma cells as signalling sources or targets. (**F**,**G**) Tile plots showing differential ligand–receptor interactions between MEI1-positive and MEI1-negative plasma cells with immune and stromal cells.

Because plasma cells were enriched in Niche 9, we examined plasma-cell heterogeneity. MEI1 was preferentially expressed in plasma cells (Figure 5A), and MEI1-positive cells showed enrichment of endoplasmic reticulum processing, protein export, N-glycosylation, immunoglobulin-associated, and B-cell receptor programmes, consistent with enhanced secretory activity (Figure 5B).

Regulon analysis identified increased SREBF2, ETV6, CREB3L2, and NFKB1 activity, with predicted targets related to inflammatory signalling, lipid metabolism, stress adaptation, and cell-cycle regulation (Figure 5C; Supplementary Figure S5A–E). MEI1-positive plasma cells also showed distinct immune-, stromal-, and tumour-associated ligand–receptor patterns and altered metabolic programmes involving fatty acid breakdown, acetyl-CoA generation, N-glycosylation, and amino acid and nucleotide metabolism (Figure 5D–G; Supplementary Figure S5F).

### 3.7. The Immune-Enriched Niche-Associated Programme Is Associated with Immunotherapy Response

**Figure 6:**
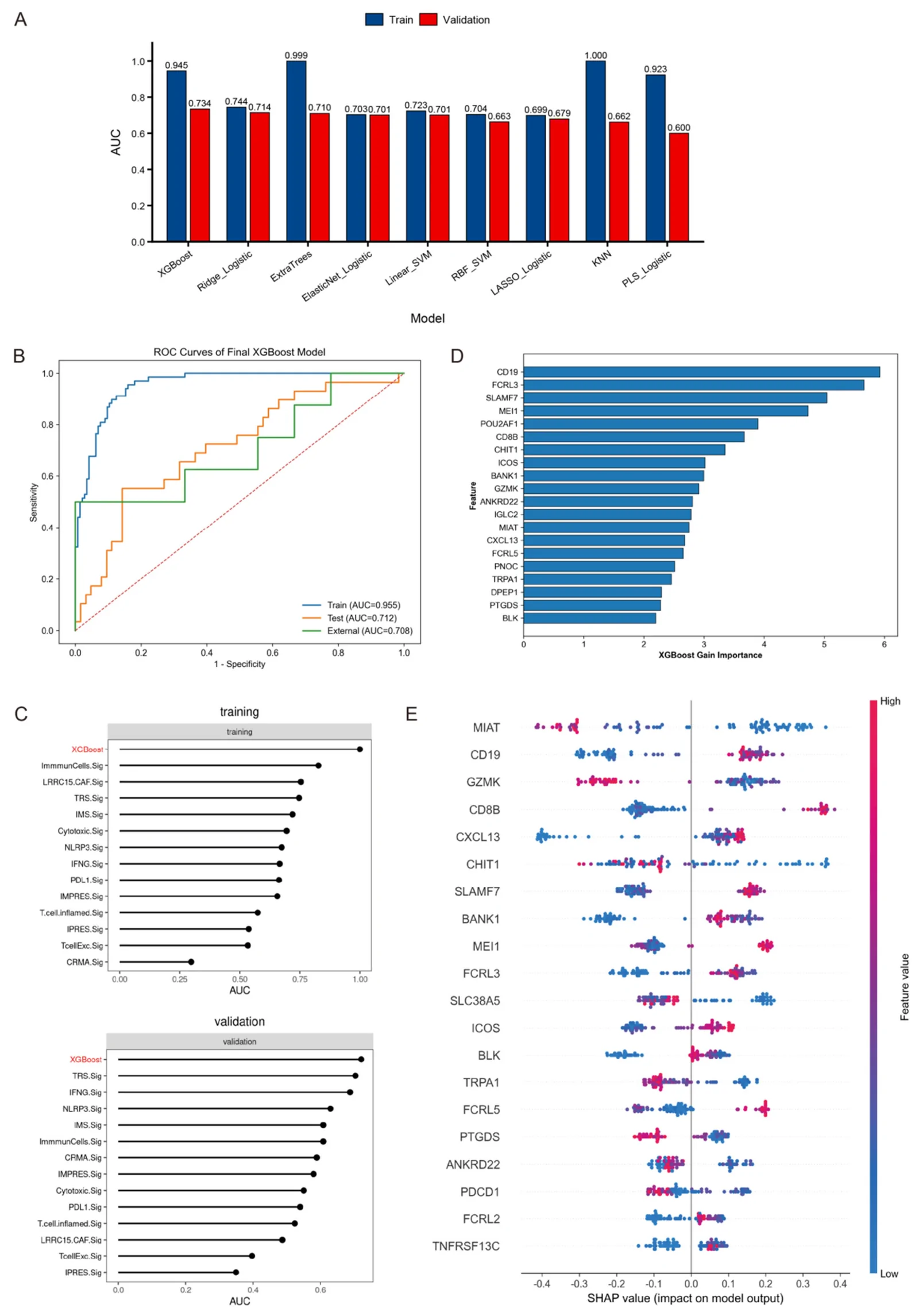
Construction and validation of a niche-derived machine-learning signature for predicting immunotherapy response in hepatocellular carcinoma. (**A**) Comparison of multiple machine-learning models identified XGBoost as the final predictive model based on overall area under the receiver operating characteristic curve performance. (**B**) Receiver operating characteristic curves showing the performance of the final XGBoost model in the training, internal test, and independent external validation cohorts. (**C**) Area under the receiver operating characteristic curve ranking of candidate predictors in the training and validation cohorts. (**D**) XGBoost feature importance analysis highlighting immune-related genes contributing to model prediction. (**E**) Shapley additive explanations summary plot showing the impact of individual genes on model output.

A Niche 9-associated programme based on the top 100 upregulated genes was evaluated using nine machine-learning algorithms in the EGAS00001005503 ATZ/BVZ-treated cohort (Figure 6A; Supplementary Table S1). XGBoost showed the best overall performance, with training and validation AUCs of 0.945 and 0.734, respectively. The final model achieved ROC-AUCs of 0.955, 0.712, and 0.708 in the training, internal test, and independent validation cohorts (Figure 6B), with performance comparable to or exceeding that of several established immunotherapy-related signatures (Figure 6C).

Feature importance and SHAP analyses highlighted immune-related genes, including CD19, CD8B, CXCL13, SLAMF7, MEI1, ICOS, FCRL5, BLK, FCRL2, and TNFRSF13C, as positive contributors to response classification (Figure 6D,E).

### 3.8. Association of the Niche-Associated Programme with Immune Features and Clinical Outcomes

**Figure 7:**
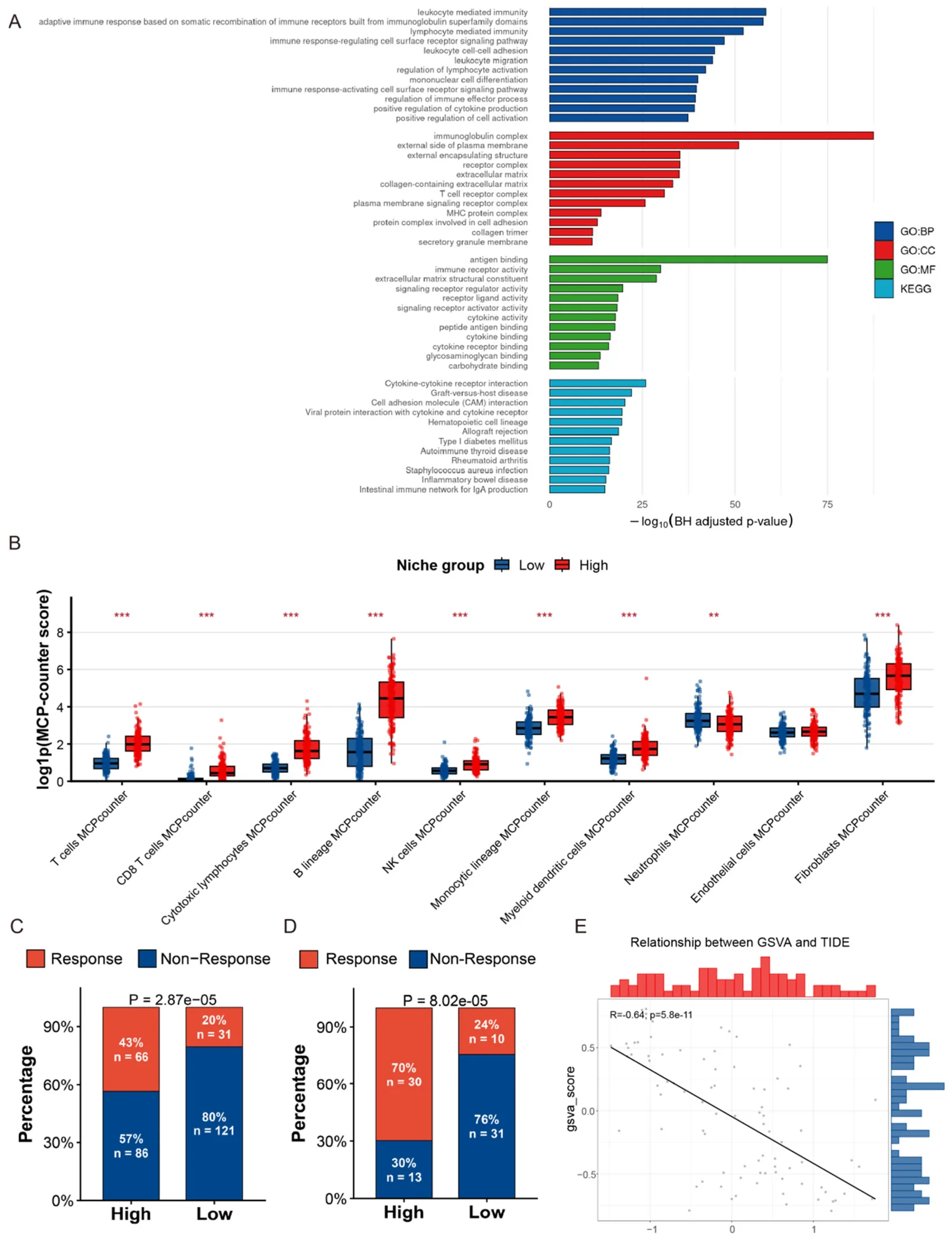
High signature scores are associated with immune activation, immune-cell infiltration, and TLS-associated immune programmes. (**A**) Functional enrichment analysis of niche-derived genes showing significant immune-related Gene Ontology and Kyoto Encyclopedia of Genes and Genomes pathways. (**B**) Comparison of immune-cell infiltration between signature-high and signature-low groups. (**C**,**D**) Response proportion analysis showing that patients with high signature scores had a higher proportion of responders in both the training and validation cohorts. (**E**) Correlation analysis between the gene set variation analysis-derived signature score and Tumour Immune Dysfunction and Exclusion score. ** *p* < 0.01,*** *p* < 0.001.

Ten core genes were selected to construct the final niche-associated transcriptional programme. High programme scores were associated with enrichment of adaptive immune pathways, including antigen recognition, cytokine signalling, B- and T-cell receptor pathways, antigen presentation, leukocyte migration, and IgA-related processes (Figure 7A).

MCP-counter analysis showed increased T-cell, cytotoxic, B-lineage, NK-cell, monocytic, and myeloid dendritic-cell signatures in programme-high tumours, with B-lineage signatures showing the strongest association (Figure 7B). GSVA further confirmed enrichment of immune-related and TLS-associated programmes (Supplementary Figure S6A). Spearman correlation analysis revealed that the 10-gene GSVA score was strongly positively correlated with B-lineage cells (ρ = 0.90), T cells (ρ = 0.87), cytotoxic lymphocytes (ρ = 0.79), CD8^+^ T cells (ρ = 0.78), myeloid dendritic cells (ρ = 0.66), and the ESTIMATE ImmuneScore (ρ = 0.83) (Supplementary Figure S6B).

Clinically, higher programme scores were associated with increased immunotherapy response rates, lower TIDE scores, and improved overall and progression-free survival (Figure 7C–E; Supplementary Figure S6C–E). In multivariable logistic regression models adjusted for T-cell-inflamed, IFN-γ response, cytotoxic lymphocyte, and curated TLS signatures, the association between the ten-gene score and ATZ/BVZ response was variably attenuated but remained evident after adjustment for several established immune programmes (Supplementary Figure S6F).

## 4. Discussion

In this study, we integrated single-cell and spatial transcriptomics, spatial proteomics, metabolic inference, cell–cell communication analysis, and machine learning to characterize spatially organized immune ecosystems in hepatocellular carcinoma (HCC). We identified an immune-enriched spatial niche, Niche 9, characterized by coordinated enrichment of B cells, plasma cells, natural killer cells, T-cell subsets, and macrophages. This transcriptional state was reproduced in an independent pretreatment spatial cohort, where Niche 9-high regions were more frequent in responders at the pooled spot level, while spatially co-registered CODEX data supported enrichment of lymphoid populations and distinct regional protein features. Together, these findings support Niche 9 as a reproducible spatial immune state rather than a dataset-specific clustering pattern.

Because the discovery cohort lacked matched atezolizumab-plus-bevacizumab (ATZ/BVZ) outcomes, Niche 9 should not be considered a spatial biomarker directly established for ATZ/BVZ response. However, its transcriptional programme was enriched in responders in an independent pretreatment nivolumab-based spatial cohort and showed consistent associations with response and survival in independent ATZ/BVZ-treated bulk cohorts. Importantly, the distinct immunomodulatory effects of VEGF blockade in ATZ/BVZ therapy may influence the spatial immune landscape and should be considered when interpreting findings across treatment regimens. Additionally, the exact Fisher‘s test results should be interpreted as descriptive findings.

As the spatial comparison was performed at the pooled spot rather than patient level, these findings should be interpreted as supportive evidence of immunotherapy relevance rather than definitive predictive validation. These findings suggest that the Niche 9-associated immune programme may capture shared features of immunotherapy-responsive tumour microenvironments across different treatment settings.

Niche 9 displayed several features associated with organized lymphoid immunity, including B-cell and plasma-cell enrichment, B-cell receptor signalling, lymphocyte co-stimulation, chemokine signalling, and TLS-related transcriptional programmes [37,38,39]. CODEX analysis further supported enrichment of B- and T-cell compartments. However, spot-based transcriptomics and regional multiplex protein measurements cannot establish canonical tertiary lymphoid structure architecture. Niche 9 is therefore better regarded as a lymphoid-enriched immune niche with TLS-associated molecular and cellular features [40,41].

Niche 9 occurred in both intratumoural and peritumoural regions and was frequently located near tumour–stromal interfaces, suggesting that local cellular organization may contribute to antigen presentation, lymphocyte recruitment, and maintenance of immune activity. Its reproducibility across spatial datasets and its lymphoid-enriched CODEX phenotype further indicate that spatial organization, rather than immune-cell abundance alone, is an important feature of this state.

Metabolic and communication analyses provided additional functional context. Niche 9 showed altered amino acid, lipid, glutathione, methionine, and choline-related programmes that may reflect the demands of activated and secretory immune populations [42]. Recurrent SLAMF7-, CD84-, CADM1-, and MMP9–CD44-associated spatial patterns suggested coordinated immune and stromal communication, although these signals should not be interpreted as direct cell-type-specific interactions because individual spatial spots contain multiple cell populations and the resolution of spatial transcriptome sequences at the level of single cells 55 micrometres in diameter cannot be reached [43,44].

MEI1-positive plasma cells exhibited transcriptional programmes related to immunoglobulin production, endoplasmic reticulum processing, protein transport, and N-glycosylation, consistent with enhanced secretory adaptation. Increased SREBF2, ETV6, CREB3L2, and NFKB1 regulon activity further suggested coordinated metabolic and stress-response regulation. Given the known reprogramming of antibody-secreting cells [45], MEI1-positive cells may represent a secretory plasma-cell state associated with humoral immune activity. However, our analyses identify MEI1 as a marker of this state and do not establish a direct regulatory role. Although our computational analyses provided insights into the potential biological features of Niche 9, the inferred metabolic activities, ligand–receptor interactions, transcriptional regulatory programmes, and functions of MEI1-positive plasma cells require further experimental validation to establish their functional relevance.

To translate the spatial findings into bulk cohorts, we derived a nine-gene Niche 9-associated signature using machine-learning feature selection and SHAP analysis. Higher scores were associated with immunotherapy response, lymphoid and B-lineage immune features, lower Tumour Immune Dysfunction and Exclusion scores, and improved survival. Because the signature is derived from bulk RNA sequencing, it should be regarded as a molecular proxy rather than a direct surrogate for Niche 9 spatial architecture. Adjustment for established T-cell-inflamed, IFN-γ response, cytotoxic lymphocyte, and TLS signatures variably attenuated its association with ATZ/BVZ response, suggesting partial overlap with established immune-active programmes while potentially retaining additional information. Its incremental predictive value requires validation in larger independent cohorts.

Several limitations should be acknowledged. The discovery cohort lacked matched ATZ/BVZ outcomes, whereas the external spatial cohort received nivolumab-based therapy. The spatial response analysis was performed at the pooled spot level, spatial cohort sizes were limited, and CODEX-derived regional protein enrichment may reflect differences in cell composition, per-cell abundance, or both. The ten-gene signature also overlaps biologically with established immune-inflammatory programmes. Nevertheless, whether this gene set provides additional predictive information beyond established immune-infiltrated signatures requires further validation in larger independent cohorts. Finally, metabolic states, ligand–receptor relationships, transcriptional programmes, and the biological role of MEI1-positive plasma cells were computationally inferred and require experimental validation.

## 5. Conclusions

In summary, we identified a reproducible lymphoid-enriched spatial immune niche in hepatocellular carcinoma with tertiary lymphoid structure-associated molecular and cellular features. This niche was characterized by coordinated lymphoid and myeloid organization, distinct metabolic and intercellular communication programs, and a MEI1-positive plasma-cell state with enhanced secretory adaptation. A Niche 9-derived transcriptional signature was further associated with atezolizumab-plus-bevacizumab response and favourable clinical outcomes in independent bulk cohorts. These findings support a link between spatial immune organization and immunotherapy sensitivity in HCC and provide a molecular framework for translating spatially defined immune states into clinically applicable transcriptomic biomarkers. Further treatment-matched spatial and functional validation will be required to establish its predictive and mechanistic relevance.

## Data Availability

The publicly available datasets analysed in this study can be obtained from the corresponding public repositories, including PRJCA007744, HRA000437, EGAS00001005503, GSE202069, and GSE285963. The independent spatial multi-omics cohort used for external Visium and CODEX validation was obtained from the publicly available Zenodo repository associated with the bioRxiv preprint by Wu et al. [30]. (2025; https://doi.org/10.1101/2025.06.11.656869). Processed data and analysis code generated during this study are available from the corresponding author upon reasonable request.

## Supplementary Materials

The following supporting information can be downloaded at https://www.mdpi.com/article/10.3390/cancers18213442/s1: Figure S1: Single-cell landscape of the hepatocellular carcinoma tumor microenvironment; Figure S2: Identification of consensus spatial niches and clinical relevance; Figure S3: Independent spatial transcriptomic and CODEX validation of the Niche 9-associated immune state; Figure S4: Shared ligand-receptor-related genes associated with immune-active cellular components and favorable clinical outcomes; Figure S5: Transcription factor activity analysis and downstream regulatory target genes of MEI1-positive plasma cells; Figure S6: Clinical relevance of the Niche 9-derived response signature; Table S1: Performance comparison of nine machine-learning algorithms for predicting treatment response in the EGAS00001005503 discovery cohort.

## Abbreviations

ATZ/BVZ	Atezolizumab plus bevacizumab
AUC	Area under the receiver operating characteristic curve
BCR	B-cell receptor
CAF	Cancer-associated fibroblast
DEG	Differentially expressed gene
FDR	False discovery rate
GO	Gene Ontology
GSVA	Gene set variation analysis
HCC	Hepatocellular carcinoma
ICI	Immune checkpoint inhibitor
KEGG	Kyoto Encyclopedia of Genes and Genomes
LR	Ligand–receptor
NK	Natural killer
OS	Overall survival
PCA	Principal component analysis
PFS	Progression-free survival
ROC	Receiver operating characteristic
Ro/e	Observed-to-expected ratio
scRNA-seq	Single-cell RNA sequencing
SHAP	Shapley additive explanations
TAM	Tumour-associated macrophage
TIDE	Tumour Immune Dysfunction and Exclusion
TLS	Tertiary lymphoid structure
TME	Tumour microenvironment
Treg	Regulatory T cell
UMAP	Uniform manifold approximation and projection
UMI	Unique molecular identifier
VEGF	Vascular endothelial growth factor
