# Supplementary material for: A Lymphoid-Enriched Spatial Immune Niche Defines an Immunotherapy-Responsive State in Hepatocellular Carcinoma
Source: Cancers (Basel). 2026 Oct 24;18(21):3442. doi: 10.3390/cancers18213442 (PMC13761897; doi:10.3390/cancers18213442)
Supplement: Supplementary file 1 [file cancers-18-03442-s001.zip › cancers-4614919-supplementary.pdf]

Supporting Information

Supplementary Figure S1. Single-cell landscape of the hepatocellular carcinoma tumor microenvironment. (A) Uniform manifold approximation and projection visualization of the integrated hepatocellular carcinoma single-cell RNA-sequencing dataset before and after batch correction using batch balanced k-nearest neighbors, colored by patient. (B) Dot plot of marker genes for T/natural killer-cell subsets, including regulatory T cells, CD4-positive T cells, naive T cells, CD8-positive T cells, natural killer cells, and gamma delta T cells. (C) Dot plot of marker genes for fibroblast subsets, including vascular/myofibroblastic cancer-associated fibroblasts, matrix cancer-associated fibroblasts, inflammatory cancer-associated fibroblasts, antigen-presenting cancer-associated fibroblasts, lipofibroblast cancer-associated fibroblasts, and E2F-associated cancer-associated fibroblasts. (D) Dot plot of marker genes for endothelial cell subsets, including vascular endothelial cells, liver sinusoidal endothelial cells, and lymphatic endothelial cells. (E) Dot plot of marker genes for monocyte subsets, including CD14-positive monocytes, CD14-positive CD16-positive monocytes, and CD16-positive monocytes. (F) Dot plot of marker genes for tumor-associated macrophage subsets, including SPP1-positive, SLC40A1-positive, APOA2-positive, FYN-positive, and IL4I1-positive tumor-associated macrophages. Dot size indicates the fraction of expressing cells, and color indicates average expression.

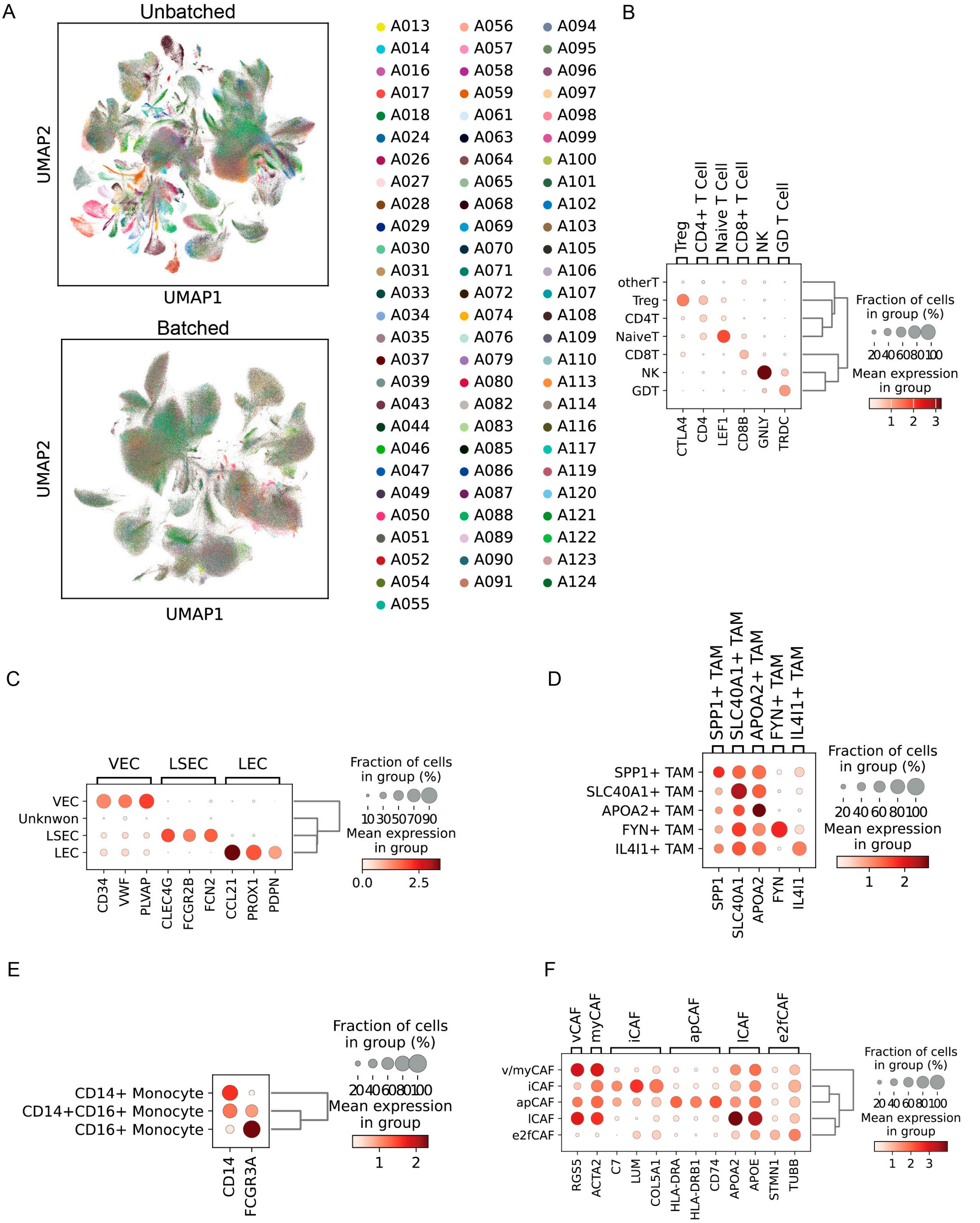

Supplementary Figure S2. Identification of consensus spatial niches and clinical relevance. (A)Hierarchical clustering of Leiden clusters based on inter-cluster similarity. (B) Pearson correlation heatmap of Leiden clusters

derived from the deconvolved cell-type abundance matrix of spatial transcriptomic spots. (C) Response rates in the immunotherapy cohort stratified by the Niche 1 signature. (D, E) Kaplan-Meier curves of overall survival and progression-free survival stratified by the Niche 1 signature. (F) Response rates in the immunotherapy cohort stratified by the Niche 3 signature. (G, H) Kaplan-Meier curves of overall survival and progression-free survival stratified by the Niche 3 signature. (I) Response rates in the immunotherapy cohort stratified by the Niche 5 signature. (J, K) Kaplan-Meier curves of overall survival and progression-free survival stratified by the Niche 5 signature.

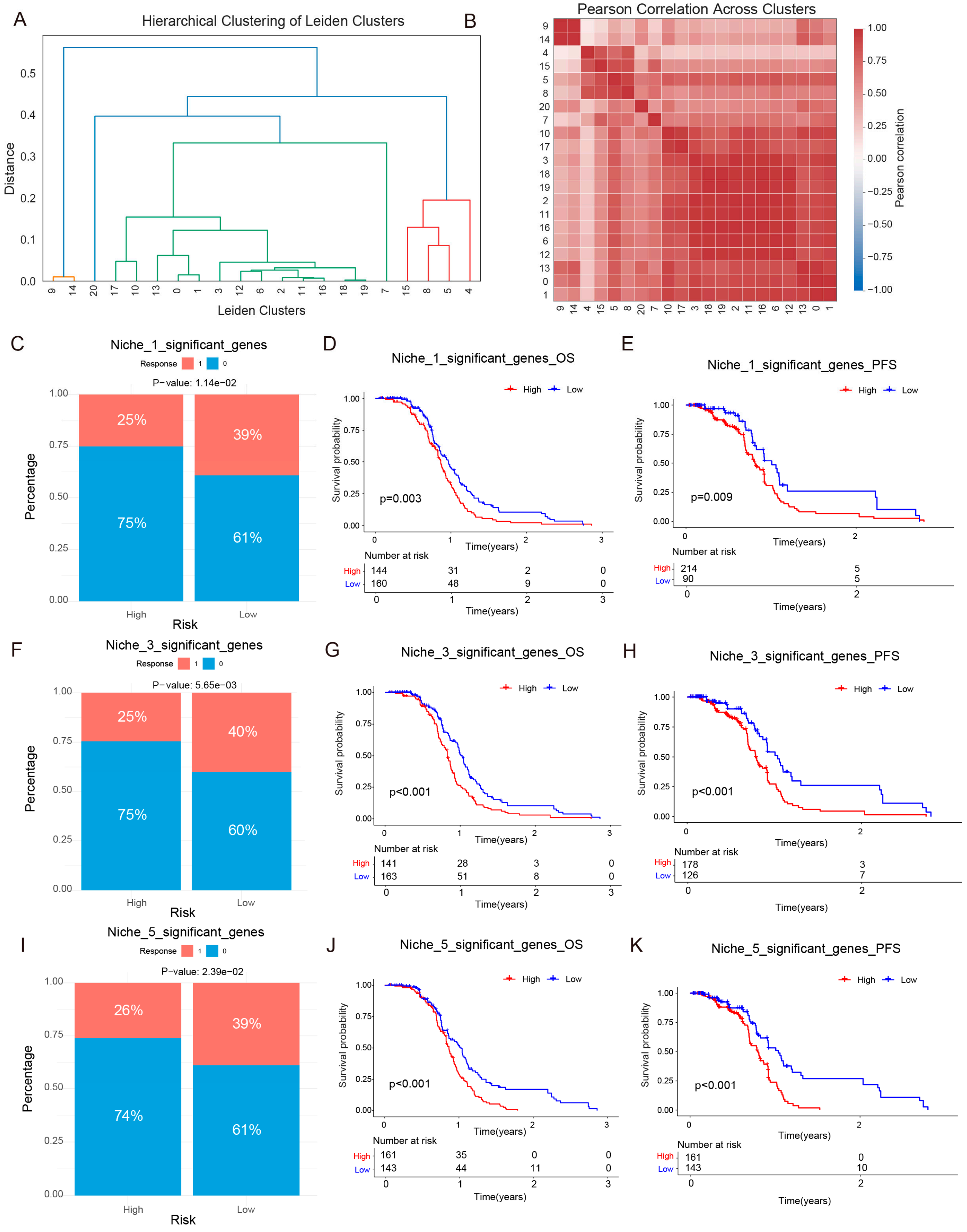
A
Hierarchical Clustering of Leiden Clusters
Distance
Leiden Clusters
B
Pearson Correlation Across Clusters
Pearson correlation
C
Niche_1_significant_genes
Response 1 0
P−value: 1.14e−02
25%
39%
75%
61%
Percentage
High
Low
Risk
D
Niche_1_significant_genes_OS
High Low
Survival probability
p=0.003
Time(years)
Number at risk
High 144 31 2 0
Low 160 48 9 0
E
Niche_1_significant_genes_PFS
p=0.009
High 214 5
Low 90 5
F
Niche_3_significant_genes
P−value: 5.65e−03
25%
40%
75%
60%
G
Niche_3_significant_genes_OS
p<0.001
High 141 28 3 0
Low 163 51 8 0
H
Niche_3_significant_genes_PFS
p<0.001
High 178 3
Low 126 7
I
Niche_5_significant_genes
P−value: 2.39e−02
26%
39%
74%
61%
J
Niche_5_significant_genes_OS
p<0.001
High 161 35 0 0
Low 143 44 11 0
K
Niche_5_significant_genes_PFS
p<0.001
High 161 0
Low 143 10

Supplementary Figure S3. Independent spatial transcriptomic and CODEX validation of the Niche 9-associated immune state.(A) Representative pretreatment spatial maps showing continuous Niche 9 AUCell scores in a responder (Patient 83) and a non-responder (Patient 73). Spots within the top 10% of Niche 9 AUCell scores are highlighted as Niche 9-high regions.(B) Proportion of Niche 9-high spots in responder and non-responder pretreatment specimens. Niche 9-high spots were more frequent in responders than in non-responders (14.8% vs 7.3%; Fisher's exact test, $P < 0.001$).(C) Associations between continuous Niche 9 AUCell activity and CODEX-derived cell-type abundance across spatially co-registered regions. Colors indicate Spearman correlation coefficients, with statistical significance assessed after false-discovery rate correction.(D) CODEX protein markers enriched in Niche 9-high regions compared with other regions. Bars indicate Cliff's delta effect sizes and are grouped into lymphoid, B-cell/TLS-associated, NK/cytotoxic, antigen-presentation/APC, immune-regulatory, and stromal-associated categories. Positive Cliff's delta values indicate higher regional protein abundance in Niche 9-high regions.*FDR, false discovery rate; APC, antigen-presenting cell; TLS, tertiary lymphoid structure. In panel C, *FDR < 0.05, **FDR < 0.01, and ***FDR < 0.001.

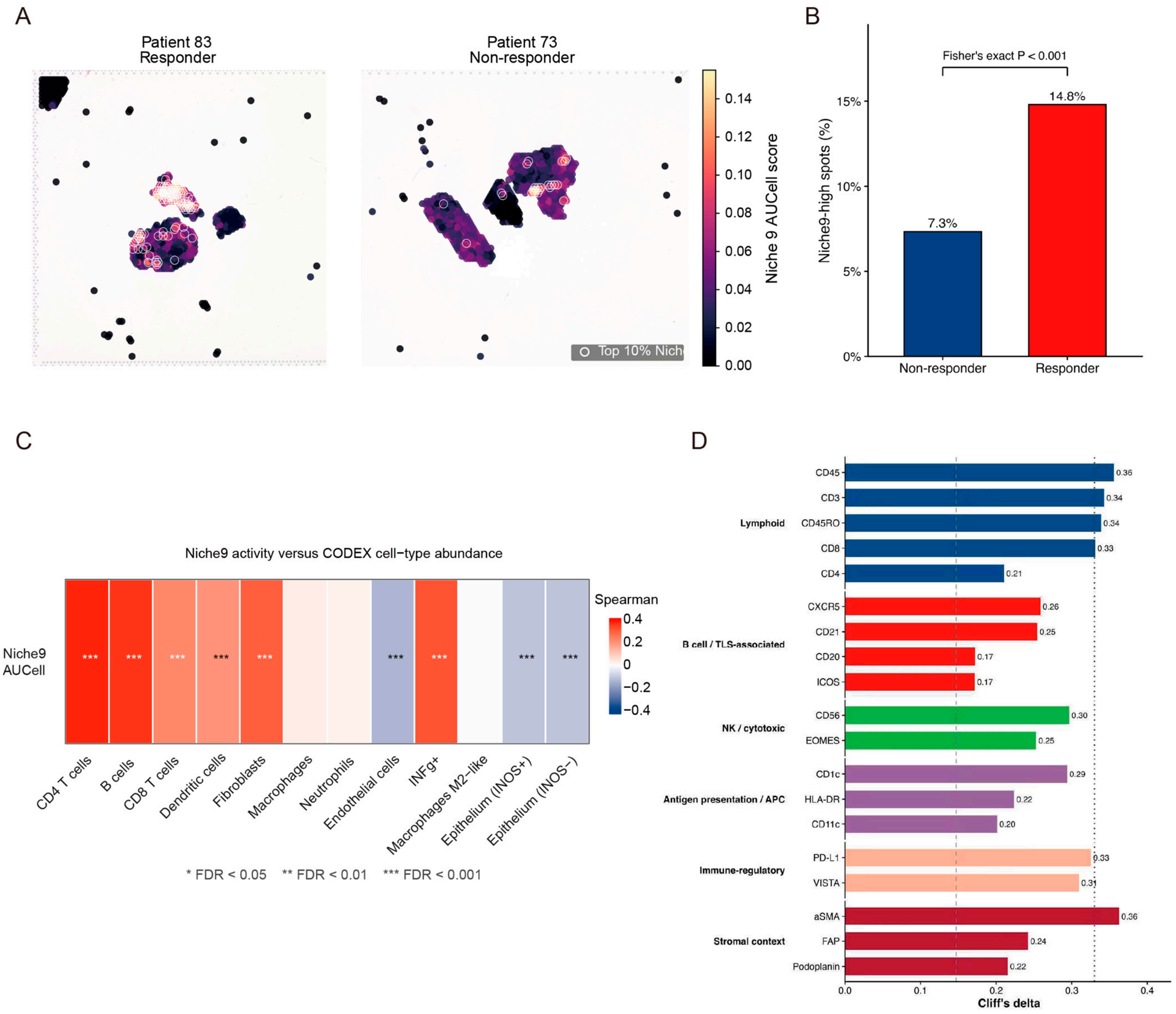

Supplementary Figure S4. Shared ligand-receptor-related genes associated with immune-active cellular components and favorable clinical outcomes. (A) Venn diagram showing the overlap between gene sets from high SLAMF7-SLAMF7 ligand-receptor score spots in HCC-2L and HCC-2T sections, identifying 145 shared genes. (B) Gene Ontology cellular component enrichment analysis of the shared genes. (C) Box plots showing the expression of SLAMF7, CD44, and MMP9 in responders and non-responders. (D) Kaplan-Meier survival curves showing the associations of SLAMF7, CD44, and MMP9 expression with progression-free survival and overall survival.

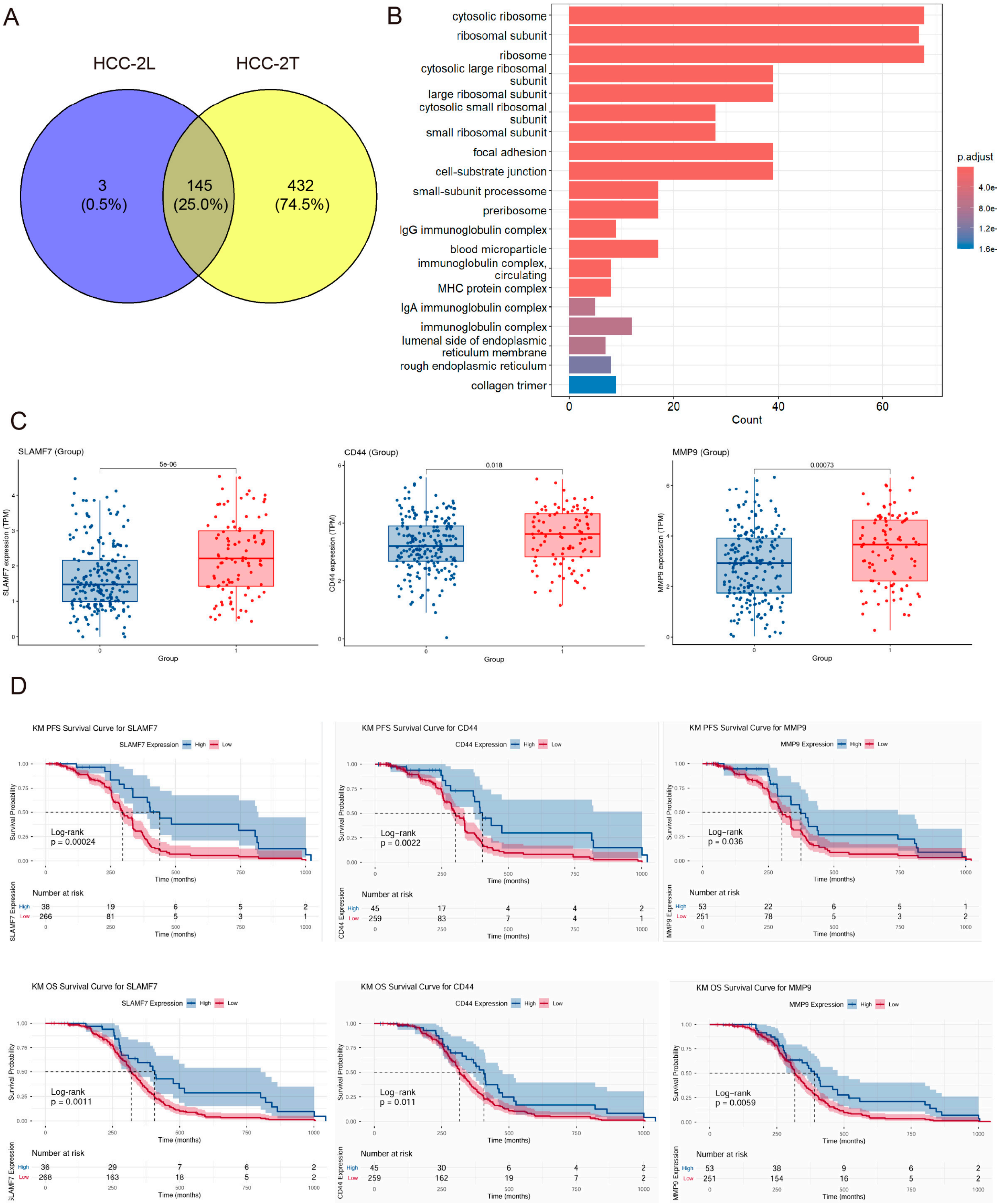
A
HCC-2L
HCC-2T
3
(0.5%)
145
(25.0%)
432
(74.5%)
B
cytosolic ribosome
ribosomal subunit
ribosome
cytosolic large ribosomal subunit
large ribosomal subunit
cytosolic small ribosomal subunit
small ribosomal subunit
focal adhesion
cell-substrate junction
small-subunit processome
preribosome
IgG immunoglobulin complex
blood microparticle
immunoglobulin complex, circulating
MHC protein complex
IgA immunoglobulin complex
immunoglobulin complex
lumenal side of endoplasmic reticulum membrane
rough endoplasmic reticulum
collagen trimer
0
20
40
60
Count
p.adjust
4.0e-08
8.0e-08
1.2e-07
1.6e-07
C
SLAMF7 (Group)
5e-06
SLAMF7 expression (TPM)
Group
CD44 (Group)
0.018
CD44 expression (TPM)
Group
MMP9 (Group)
0.00073
MMP9 expression (TPM)
Group
D
KM PFS Survival Curve for SLAMF7
SLAMF7 Expression High Low
Survival Probability
Log-rank
p = 0.00024
Time (months)
Number at risk
High 38 19 6 5 2
Low 266 81 5 3 1
KM PFS Survival Curve for CD44
CD44 Expression High Low
Log-rank
p = 0.0022
Number at risk
High 45 17 4 4 2
Low 259 83 7 4 1
KM PFS Survival Curve for MMP9
MMP9 Expression High Low
Log-rank
p = 0.036
Number at risk
High 53 22 6 5 1
Low 251 78 5 3 2
KM OS Survival Curve for SLAMF7
SLAMF7 Expression High Low
Log-rank
p = 0.0011
Number at risk
High 36 29 7 6 2
Low 268 163 18 5 2
KM OS Survival Curve for CD44
CD44 Expression High Low
Log-rank
p = 0.011
Number at risk
High 45 30 6 4 2
Low 259 162 19 7 2
KM OS Survival Curve for MMP9
MMP9 Expression High Low
Log-rank
p = 0.0059
Number at risk
High 53 38 9 6 2
Low 251 154 16 5 2

Supplementary Figure S5. Transcription factor activity analysis and downstream regulatory target genes of MEI1-positive plasma cells. (A) Violin plots showing regulon activity of ETV6, SREBF2, CREB3L2, and NFKB1 in MEI1-positive and MEI1-negative plasma cells. (B) Uniform manifold approximation and projection visualization of MEI1-positive and MEI1-negative plasma cells. Red dots indicate MEI1-positive plasma cells, and gray dots indicate MEI1-negative plasma cells. (C) Heatmap showing leading-edge gene activity across transcription factors. (D, E) Dot plots showing WikiPathways and Reactome enrichment of leading-edge genes from selected transcription factor regulons. Dot size indicates gene count, and color indicates -log10 adjusted P value. (F) Heatmap showing inferred metabolic pathway activity in MEI1-positive and MEI1-negative plasma cells. Values represent row-scaled mean flux scores.

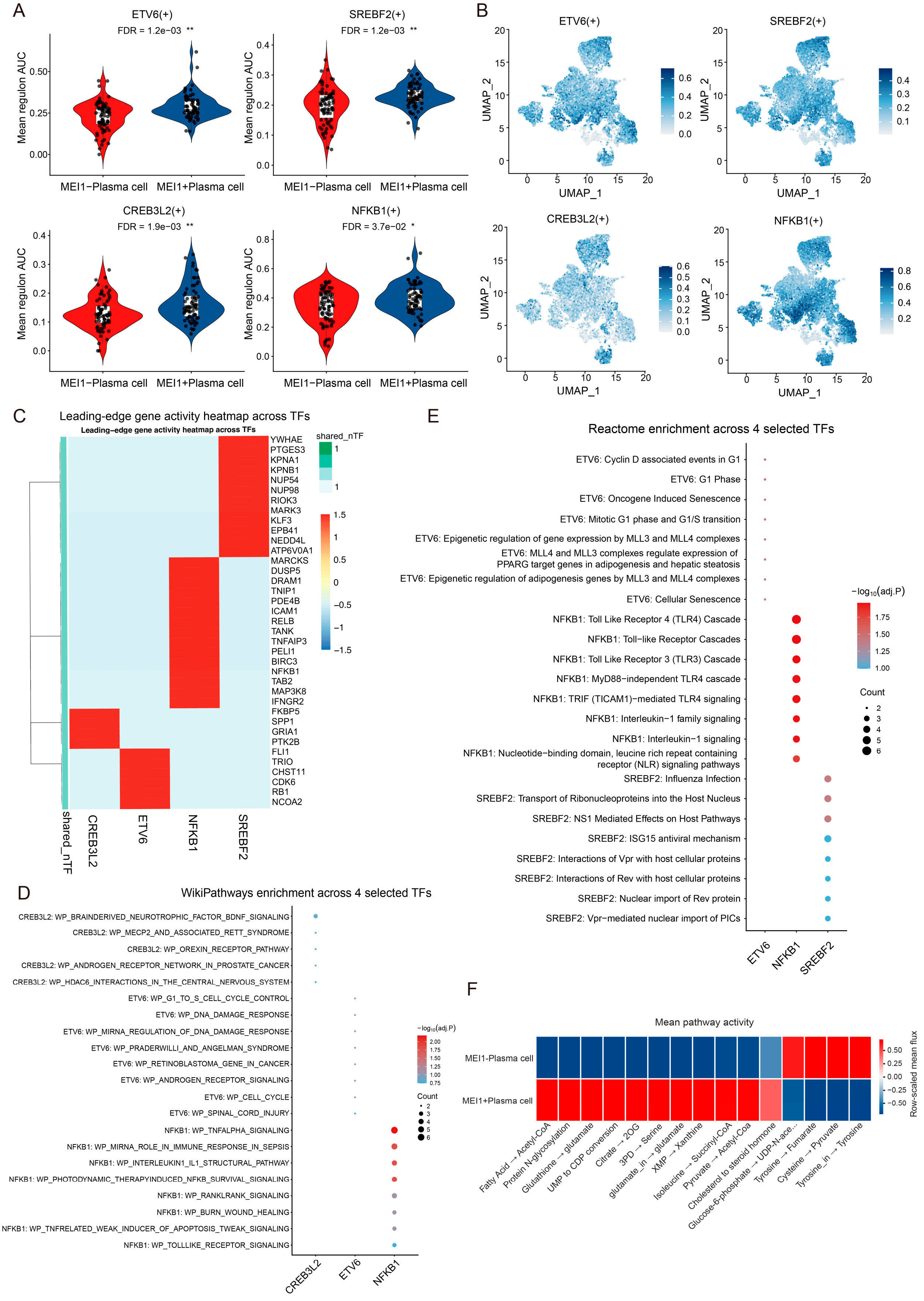
A
ETV6(+)
FDR = 1.2e−03 **
SREBF2(+)
FDR = 1.2e−03 **
CREB3L2(+)
FDR = 1.9e−03 **
NFKB1(+)
FDR = 3.7e−02 *
Mean regulon AUC
MEI1−Plasma cell
MEI1+Plasma cell
B
ETV6(+)
SREBF2(+)
CREB3L2(+)
NFKB1(+)
UMAP_1
UMAP_2
C
Leading-edge gene activity heatmap across TFs
Leading−edge gene activity heatmap across TFs
shared_nTF
YWHAE
PTGES3
KPNA1
KPNB1
NUP54
NUP98
RIOK3
MARK3
KLF3
EPB41
NEDD4L
ATP6V0A1
MARCKS
DUSP5
DRAM1
TNIP1
PDE4B
ICAM1
RELB
TANK
TNFAIP3
PELI1
BIRC3
NFKB1
TAB2
MAP3K8
IFNGR2
FKBP5
SPP1
GRIA1
PTK2B
FLI1
TRIO
CHST11
CDK6
RB1
NCOA2
CREB3L2
ETV6
NFKB1
SREBF2
D
WikiPathways enrichment across 4 selected TFs
CREB3L2: WP_BRAINDERIVED_NEUROTROPHIC_FACTOR_BDNF_SIGNALING
CREB3L2: WP_MECP2_AND_ASSOCIATED_RETT_SYNDROME
CREB3L2: WP_OREXIN_RECEPTOR_PATHWAY
CREB3L2: WP_ANDROGEN_RECEPTOR_NETWORK_IN_PROSTATE_CANCER
CREB3L2: WP_HDAC6_INTERACTIONS_IN_THE_CENTRAL_NERVOUS_SYSTEM
ETV6: WP_G1_TO_S_CELL_CYCLE_CONTROL
ETV6: WP_DNA_DAMAGE_RESPONSE
ETV6: WP_MIRNA_REGULATION_OF_DNA_DAMAGE_RESPONSE
ETV6: WP_PRADERWILLI_AND_ANGELMAN_SYNDROME
ETV6: WP_RETINOBLASTOMA_GENE_IN_CANCER
ETV6: WP_ANDROGEN_RECEPTOR_SIGNALING
ETV6: WP_CELL_CYCLE
ETV6: WP_SPINAL_CORD_INJURY
NFKB1: WP_TNFALPHA_SIGNALING
NFKB1: WP_MIRNA_ROLE_IN_IMMUNE_RESPONSE_IN_SEPSIS
NFKB1: WP_INTERLEUKIN1_IL1_STRUCTURAL_PATHWAY
NFKB1: WP_PHOTODYNAMIC_THERAPYINDUCED_NFKB_SURVIVAL_SIGNALING
NFKB1: WP_RANKLRANK_SIGNALING
NFKB1: WP_BURN_WOUND_HEALING
NFKB1: WP_TNFRELATED_WEAK_INDUCER_OF_APOPTOSIS_TWEAK_SIGNALING
NFKB1: WP_TOLLLIKE_RECEPTOR_SIGNALING
Count
E
Reactome enrichment across 4 selected TFs
ETV6: Cyclin D associated events in G1
ETV6: G1 Phase
ETV6: Oncogene Induced Senescence
ETV6: Mitotic G1 phase and G1/S transition
ETV6: Epigenetic regulation of gene expression by MLL3 and MLL4 complexes
ETV6: MLL4 and MLL3 complexes regulate expression of PPARG target genes in adipogenesis and hepatic steatosis
ETV6: Epigenetic regulation of adipogenesis genes by MLL3 and MLL4 complexes
ETV6: Cellular Senescence
NFKB1: Toll Like Receptor 4 (TLR4) Cascade
NFKB1: Toll−like Receptor Cascades
NFKB1: Toll Like Receptor 3 (TLR3) Cascade
NFKB1: MyD88−independent TLR4 cascade
NFKB1: TRIF (TICAM1)−mediated TLR4 signaling
NFKB1: Interleukin−1 family signaling
NFKB1: Interleukin−1 signaling
NFKB1: Nucleotide−binding domain, leucine rich repeat containing receptor (NLR) signaling pathways
SREBF2: Influenza Infection
SREBF2: Transport of Ribonucleoproteins into the Host Nucleus
SREBF2: NS1 Mediated Effects on Host Pathways
SREBF2: ISG15 antiviral mechanism
SREBF2: Interactions of Vpr with host cellular proteins
SREBF2: Interactions of Rev with host cellular proteins
SREBF2: Nuclear import of Rev protein
SREBF2: Vpr−mediated nuclear import of PICs
Count
F
Mean pathway activity
MEI1-Plasma cell
MEI1+Plasma cell
Fatty Acid → Acetyl-CoA
Protein N-glycosylation
Glutathione → glutamate
UMP to CDP conversion
Citrate → 2OG
3PD → Serine
glutamate_in → glutamate
XMP → Xanthine
Isoleucine → Succinyl-CoA
Pyruvate → Acetyl-CoA
Cholesterol to steroid hormone
Glucose-6-phosphate → UDP-N-ace...
Tyrosine → Fumarate
Cysteine → Pyruvate
Tyrosine_in → Tyrosine
Row-scaled mean flux

Supplementary Figure S6. Clinical relevance of the Niche 9-derived response signature. (A) Heatmap showing gene set variation analysis enrichment of tertiary lymphoid structure- and immune-related pathways across response and non-response groups. (B) Signature scores across clinical response categories assessed using the Kruskal-Wallis test. (C, D) Kaplan-Meier analysis showing overall survival and progression-free survival in patients stratified by high and low response signature scores. (E) Forest plot showing adjusted odds ratios for the standardized Niche 9 nine-gene score in multivariable logistic regression models separately adjusted for T-cell-inflamed, IFN-γ response, cytotoxic lymphocyte, and curated tertiary lymphoid structure signatures. Points indicate adjusted odds ratios and horizontal lines indicate 95% confidence intervals.

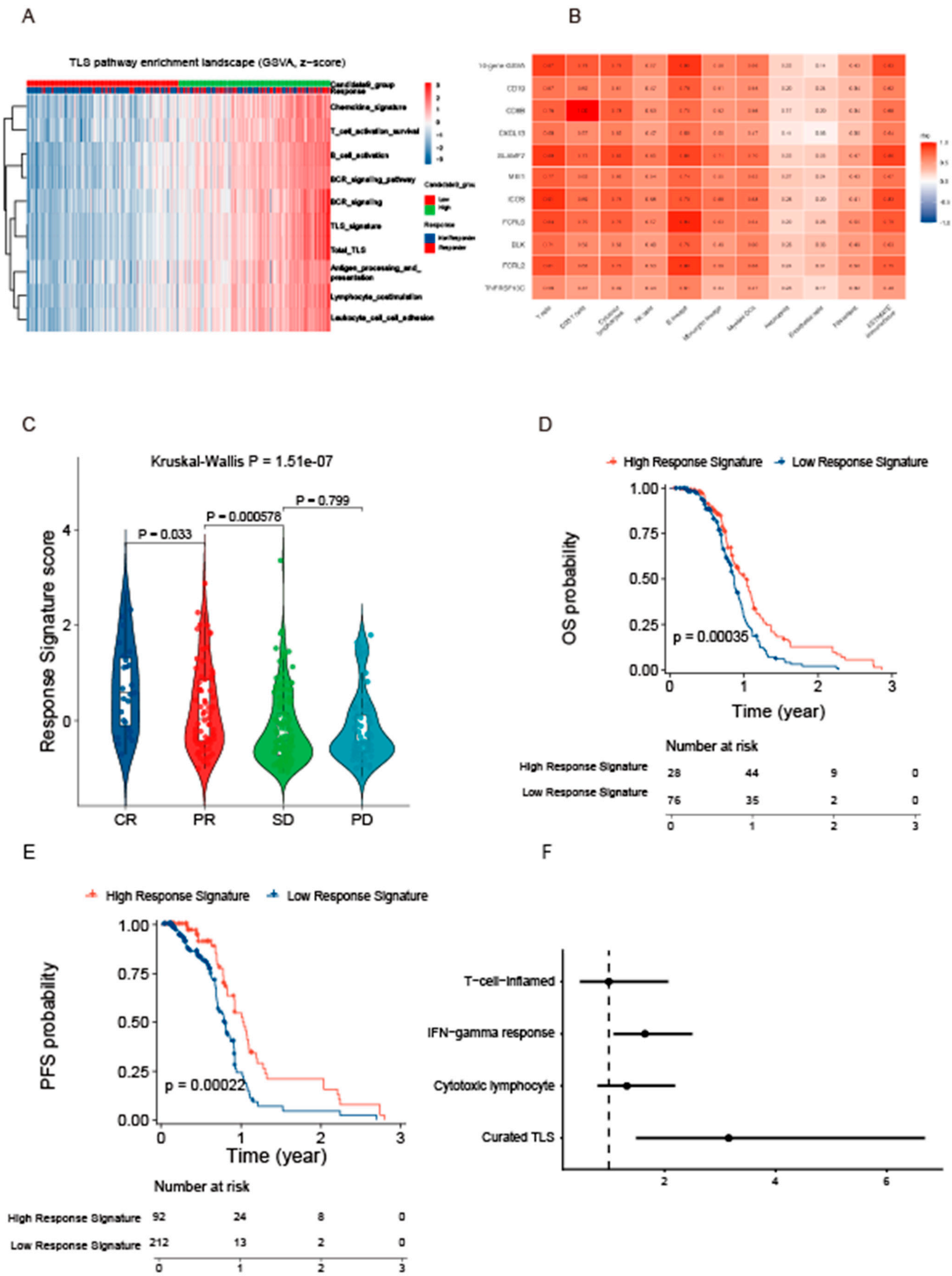
A
B
TLS pathway enrichment landscape (GSVA, z-score)
Chemokine_signature
T_cell_activation_survival
B_cell_activation
BCR_signaling_pathway
BCR_signaling
TLS_signature
Total_TLS
Antigen_processing_and_presentation
Lymphocyte_costimulation
Leukocyte_cell_cell_adhesion
C
Kruskal-Wallis P = 1.51e-07
P = 0.033
P = 0.000578
P = 0.799
Response Signature score
CR
PR
SD
PD
D
High Response Signature
Low Response Signature
OS probability
p = 0.00035
Time (year)
Number at risk
High Response Signature 28 44 9 0
Low Response Signature 76 35 2 0
E
High Response Signature
Low Response Signature
PFS probability
p = 0.00022
Time (year)
Number at risk
High Response Signature 92 24 8 0
Low Response Signature 212 13 2 0
F
T−cell−inflamed
IFN−gamma response
Cytotoxic lymphocyte
Curated TLS

Supplementary Table S1. Performance comparison of nine machine-learning algorithms for predicting treatment response in the EGAS00001005503 discovery cohort.

Model	Train_AUC	Validation_AUC	Train_Accuracy	Validation_Accuracy	Train_Precision	Validation_Precision	Train_Recall	Validation_Recall	Train_F1	Validation_F1
XGBoost	0.945	0.735	0.858	0.685	0.716	0.500	0.926	0.586	0.808	0.540
Ridge_Logistic	0.744	0.714	0.675	0.663	0.494	0.472	0.647	0.586	0.561	0.523
ExtraTrees	0.999	0.710	0.991	0.696	0.971	0.522	1.000	0.414	0.986	0.462
ElasticNet_Logistic	0.703	0.701	0.660	0.663	0.473	0.471	0.515	0.552	0.493	0.508
Linear_SVM	0.723	0.701	0.703	0.728	0.619	0.833	0.191	0.172	0.292	0.286
RBF_SVM	0.704	0.683	0.726	0.685	0.679	0.500	0.279	0.138	0.396	0.216
LASSO_Logistic	0.699	0.679	0.660	0.674	0.476	0.486	0.588	0.586	0.526	0.531
KNN	1.000	0.662	1.000	0.707	1.000	0.542	1.000	0.448	1.000	0.491
PLS_Logistic	0.923	0.600	0.858	0.587	0.732	0.355	0.882	0.379	0.800	0.367
